# 4R-tau isoform induction via TDP-43 in neurons in response to insulin: converging signaling pathways with implications for neurodegenerative disease

**DOI:** 10.1186/s40478-025-02174-x

**Published:** 2025-12-24

**Authors:** Carina Weissmann, Libia Catalina Salinas Castellanos, Mayra Micaela Montes, Gokhan Uruk, Hossam Youssef, R. Ross Reichard, Rodolfo Gabriel Gatto, Keith A Josephs

**Affiliations:** 1 IFIBYNE-UBA-CONICET Buenos Aires, Buenos Aires, Argentina; 2 Department of Neurology, Mayo Clinic, Rochester, MN USA; 3 Department of Laboratory Medicine and Pathology, Mayo Clinic, Rochester, MN USA

**Keywords:** Tau splicing, TDP-43, 3R/4R tau ratio, Insulin, Alzheimer´s disease, Corticobasal degeneration

## Abstract

Tau protein isoforms, regulated during development, are influenced by the nuclear factor TDP-43, which plays a crucial role in tau mRNA stability and exon 10 inclusion. Both tau and TDP-43 are prone to pathological phosphorylation and aggregation, with specific phosphorylated forms of TDP-43 linked to cytoplasmic mislocalization and alterations in the 3R/4R tau ratio as detected in different pathologies. In this study, we show that insulin treatment of embryonic mouse primary cortical neurons—cells that normally express only 3R-tau—induces the expression of 4R-tau, suggesting that metabolic signaling can influence tau isoform expression in a developmentally immature neuronal context. In addition, experiments in HEK293 cells revealed isoform-specific stabilization effects and showed that insulin promotes TDP-43 redistribution to the cytoplasm along with a phosphorylation pattern. These results underscore the complex interplay between TDP-43 and tau isoforms and metabolic signaling pathways that play a crucial role in their expression and localization with potential implications for understanding mechanisms of neurodegenerative disease onset and progression.

**Graphical abstract:**

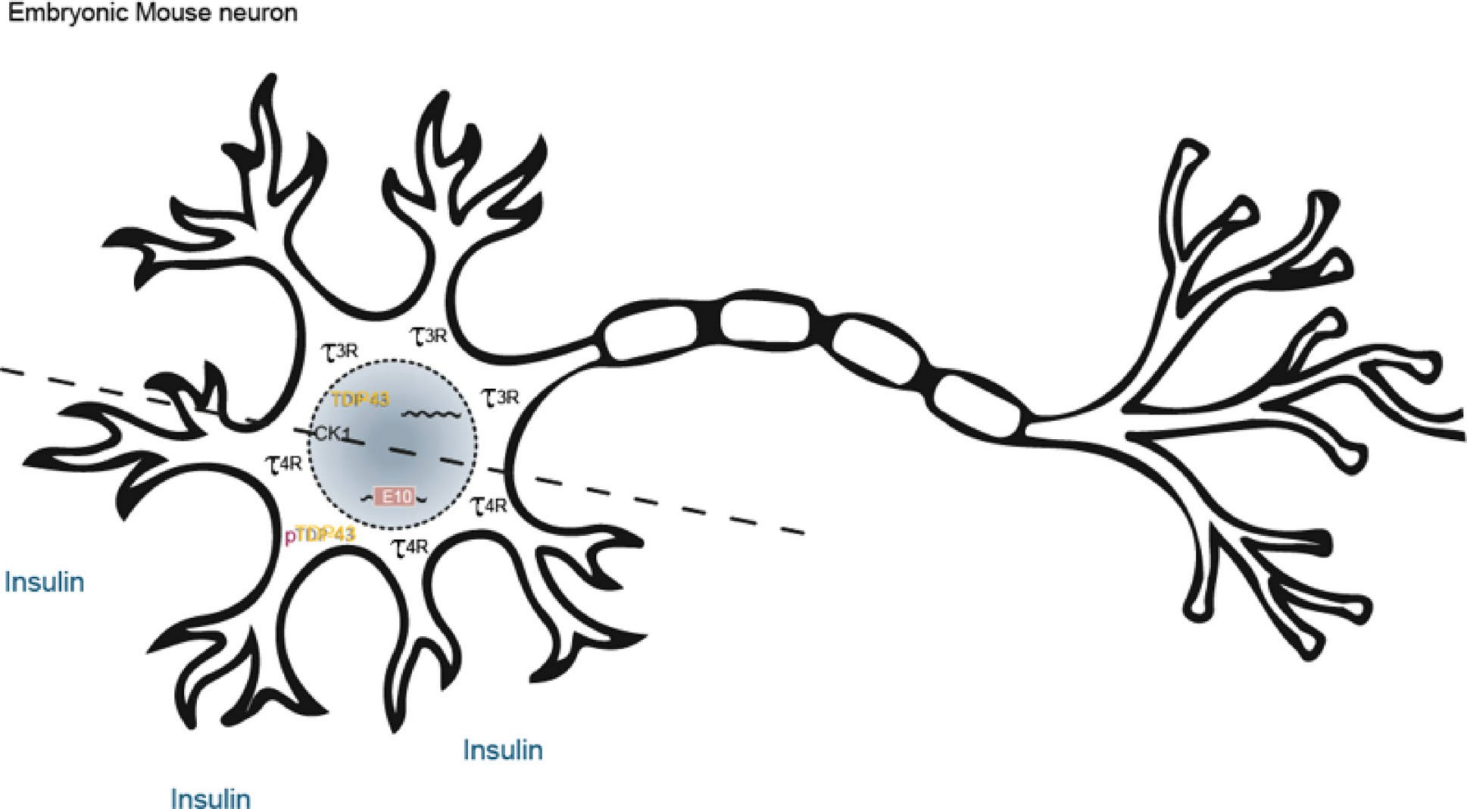
Schematic representation of an embryonic mouse neuron illustrating key molecular changes described in this study. In the untreated state (upper part), the neuron expresses 3R-tau and tau mRNA lacking exon 10 (E10), with TDP-43 localized predominantly in the nucleus. Upon insulin stimulation (lower part), 4R-tau expression is induced along with tau mRNA including E10, and phosphorylated TDP-43 (pTDP-43) is redistributed toward the somatic compartment. CK1 is shown as the kinase responsible for TDP-43 phosphorylation, mediating the downstream events observed.

**Supplementary Information:**

The online version contains supplementary material available at 10.1186/s40478-025-02174-x.

## Introduction

Neurodegenerative diseases (NDs) represent a growing global health burden, with the population over 60 expected to more than double by 2050 [1]. Among them, Alzheimer’s disease (AD) is the most prevalent, accounting for the majority of dementia cases in the elderly [2]. The neuropathological hallmarks of AD—amyloid beta (Aβ) plaques and intracellular tau neurofibrillary aggregates—have long been central to our understanding of disease progression. However, increasing evidence points to the involvement of additional proteins and mechanisms, including dysregulated RNA processing and metabolic dysfunction.

Tau is encoded by the microtubule-associated protein tau (*MAPT*) gene, located on chromosome 17q21.3 and comprising 16 exons. Alternative splicing of exons 2, 3, and 10 in MAPT pre-mRNA generates six isoforms in the human brain, with protein lengths ranging from 352 to 441 amino acids. Inclusion (4R) or exclusion (3R) of exon 10 determines the number of microtubule-binding repeats [3]. A balanced 3R/4R tau ratio is believed to be essential for normal microtubule function [4], and an imbalance has been detected in several tauopathies [3, 5, 6]. These disorders can be classified based on the predominant tau isoform in inclusion bodies—3R, 4R, or a balanced 3R:4R ratio [5].

In AD, both 3R and 4R tau isoforms are found in inclusions, though the precise balance and pathological relevance of each remain debated. In advanced AD stages, regional differences in isoform expression have been observed. For example, in brain regions with abundant extracellular neurofibrillary tangles (NFTs), such as the subiculum, CA1, and superficial layers of the entorhinal cortex, NFTs are often immunostained exclusively with 3R-specific antibodies [6]. Similarly, Yasojima et al. reported an increase 4R/3R tau ratio [4].

While adult human brains express both isoforms in roughly equal proportions, rodent models predominantly express 3R-tau during embryonic development and switch to 4R tau postnatally [7]. Differences between human and mouse tau proteins exist, particularly in the N-terminal region [8]. Nevertheless, transgenic mouse models have been widely used in tauopathy research. Many studies have utilized the human tau mouse model, which expresses the full-length human MAPT gene and produces all six human tau isoforms in the adult brain, with a bias toward 3R over 4R isoform production. Studies have reported a skewed ratio may underlie the tau pathology and associated phenotypes observed in aged human tau mice, including hyperphosphorylation and cognitive deficits. Interestingly, a trans-splicing strategy to reduce total tau expression prevented these phenotypes [9]. However, recent studies revealed that transgene insertions can disrupt endogenous gene sequences, leading to structural variations and possible deletions at the insertion site [9]. Although the effects on tauopathy pathogenesis are not fully understood, these disruptions may influence age-related neurodegeneration, including tangle formation and brain atrophy that may confound interpretation of disease mechanisms in this model.

Recent evidence also implicates TAR DNA binding protein 43 (TDP-43), a DNA/RNA-binding protein, in the regulation of tau pre-mRNA splicing, particularly through modulation of exon 10 inclusion, which determines the production of the 4R isoform [10]. TDP-43 was first associated with neurodegenerative diseases in 2006 by Neumann et al. and Arai et al. [11], when the protein was identified as a major protein component in insoluble inclusions of patients with Frontotemporal Lobal Degeneration with Ubiquitin-positive inclusions (FTLD-U) and Amyotrophic Lateral Sclerosis (ALS) patients [12].

Beyond RNA-binding proteins, metabolic signaling pathways have been implicated in tau pathology. In AD, brain hypometabolism can precede the appearance of clinical symptoms by more than a decade [13]. Impaired insulin signaling, a hallmark of AD [14], leads to disrupted glucose metabolism and has been proposed to contribute to disease progression, giving rise to the concept of AD as “type 3 diabetes” [15]. Insulin resistance is associated with increased tau phosphorylation and altered isoform expression. Decreased insulin levels in the cerebrospinal fluid (CSF) of AD patients, likely due to peripheral hyperinsulinemia and reduced transport across the blood–brain barrier (BBB), further complicate this relationship [13]. Type 2 diabetes mellitus (T2DM) increases the risk of developing AD, and conversely, AD patients often present with glucose intolerance and elevated plasma insulin levels [16]. In a chronic T2DM, Jung et al. observed an increase in 3R-tau expression, which contributed to AD-like tau pathology [16]. With disease progression, elevated 3R-tau levels have been documented, further supporting a link between metabolic dysfunction and tau isoform imbalance.

In this study, we investigated the influence of insulin signaling on tau isoform regulation. We used embryonic mouse primary cortical and hippocampal neurons to examine changes in tau splicing, while HEK293 cells were also employed to explore associated molecular pathways, including protein stability and subcellular redistribution. Our findings point to a potential link between metabolic cues and tau isoform dynamics, including possible effects on TDP-43 localization and modification. These results support a mechanistic connection between insulin signaling and tau splicing regulation, offering new insights into the pathophysiology of isoform imbalances in neurodegenerative diseases.

## Materials and methods

### Cellular and molecular biology

Human embryonic kidney 293 (HEK) cells [passage 18–26, American Type Culture Collection (ATCC) number CRL-1573] were maintained by serial passages.

Primary hippocampal and cortical cultures were prepared from mice of the C57BL/6 genetic background and prepared according to the protocol used in [17, 18]. In brief, Neurons were grown in Neurobasal medium™ (Thermo Fisher) with B27 supplement (Thermo Fisher), glutamine (Thermo Fisher) and Pen/Strep (Thermo Fisher) and used after 7–8 days in vitro. All experiments involving mice were performed following national guidelines for the humane treatment of laboratory animals from University of Buenos Aires (approved protocol # 112), which are comparable to those of the USA National Institutes of Health.

For biochemical and molecular analysis, 6 or 12-plates were coated with 0.1 mg/mL of poly-L-lysine (PLL, Sigma, P2636) and HEK cells were plated at a density of 2.2 × 10^5^ or 1.4 × 10^5^ respectively. HEK cells were grown in Dulbecco’s Modified Eagle’s Medium containing 4 mM L-glutamine, 4.5 g/L glucose, and 110 mL/L sodium pyruvate and supplemented with 10% Fetal Calf Serum (NatoCor) (or without serum) kept at 37 °C and under 5% CO2.

Notice that the glucose concentration in both DMEM and Neurobasal™ media (approximately 17.5–25 mM and 25 mM, respectively) is considerably higher than physiological plasma levels (~ 5 mM). However, since all experimental groups were exposed to the same culture conditions and only drug treatments varied, observed effects can be attributed to the specific pharmacological interventions.

### Transfections and plasmids

Cells were transfected using the calcium phosphate method as described previously [8]. The plasmids used included mCherry-TDP-43, a kind gift from Dr. Weihl (Washington University) [16]; tau2N4R-eGFP (full-length tau 441-WT fused to eGFP), a kind gift from Dr. Bloom (University of Virginia); tau2N4R-P301L-ECFP, a full-length tau construct carrying the P301L mutation fused to eCFP, provided by Dr. Gerold Schmitt-Ulms (University of Toronto); and tau1–255-eGFP, encoding a truncated tau isoform (amino acids 1–255) fused to eGFP, also from Dr. Schmitt-Ulms. All plasmids were under the control of the CMV promoter. Cells were processed for analysis 48 h post-transfection.

### Treatments and drugs

For treatments, insulin (**Ins**) was used at 50 nM for 60 min, although the concentration of insulin used in our study (50 nM) exceeds the physiological levels typically reported in the central nervous system (in the picomolar range), it is consistent with concentrations commonly employed in neuronal culture studies to ensure robust activation of insulin signaling pathways [19]. To further assess the concentration dependency, cells were also exposed to lower insulin concentrations (0.1–0.5 nM; Supplementary Fig. 1C). These conditions elicited detectable but progressively reduced responses, consistent with a dose-dependent effect.; mouse **NGF** (nerve growth factor 2.5 S Alomone N-100 ) at 100 ng/ml for 60 min as in [20]; Okadaic Acid (**OA**) as PP1a and PP2a 10 nM and 100 nM 24 h before incubation with insulin as used in [21] ; CK1 inhibitor **D4776** (Medchemexpress, stock 1 mg/25 ml DMSO) 100 µM and 1000 µM 1 h before other treatment was used at the following concentrations before incubation with insulin as used in [22]; CK2 inhibitor **DMAT** (Medchemexpress, HY-15535, (stock 1 mg/DMSO) 20 µM and 200 µM, 24 h incubation before other treatment, as previously used in [23].

### Cell processing

For whole-cell lysate (WCL) preparation, cells were washed once in PBS and resuspended in a 1% SDS HEPES pH7.4 lysis buffer containing a protease inhibitor cocktail (Roche, cOmplete™) with or without the addition of phosphatase inhibitors: NaF (10mM) and Na_3_VO_4_ (2mM), as stated in the text, and sonicated (Fisher Scientific Sonic Dismembrator, model 500) at a 20% amplitude for a 15 s pulse.

For microscopy experiments, cells were plated on glass coverslips (12 mm rounded Carolina^®^ Assistant-Brand Cover), coated with 1 mg/mL of PLL (Sigma, P2636).

### Western blotting (WB)

Proteins were resolved by either 4–10% polyacrylamide or 4–8% polyacrylamide (“mobility”) gels and transferred onto Immobilon^®^-FL PVDF membranes (BioRad). Non-specific binding was blocked by 1% non-fat powdered milk in TBS containing 0.2% Tween-20 for 60 min at RT. Membranes were incubated overnight at 4 °C with primary antibodies in 1% BSA TBS, followed by the addition of horseradish peroxidase-conjugated secondary antibodies in 1% non-fat powdered milk in TBS. Immunoreactive bands were detected by the LI-COR Odyssey system, using secondary antibodies: 926-68073 IRDye 680RD Donkey anti-Rabbit IgG or 926-32212 IRDye 800CW Donkey anti-Mouse. In mobility gel, molecular weight markers do not migrate consistently and therefore were not included.

The following primary antibodies were used: mouse monoclonal anti-**4R-Tau** (Millipore, 05-804, 1:1000); mouse monoclonal anti-**tubulin** (DM1a) (Cell Signaling #3873, 1:5000); rabbit polyclonal anti-**total tau** (Santa Cruz, sc-390476, 1:1000); anti **pTDP-43** (Cosmo Bio TIP-PTD-M01, 1:1000, Jacksonville, FL); mouse monoclonal **TDP-43** (Cell Signaling, #3449, 1:1000); **pSer** (Stressmarq, SPC-149, 1:1000 ); Rabbit anti-**mCherry** (Cell Signaling, #43590; 1:1000); mouse anti-**GFP** (Cell Signaling, #2955; 1:1000).

Images were taken using the Fuji AI600 imaging system or LI-COR Odyssey system and quantified with ImageJ software (NIH, USA).

*Detection of proteins by immunofluorescence (IF).*

Cells grown on PLL-coated glass coverslips were fixed with 4% p-formaldehyde in PBS, permeabilized with 0.1% Triton X-100 (10 min), and treated with blocking solution (1% BSA, 0.01% Triton X-100 in PBS) for an hour at RT. Coverslips were then incubated with the primary antibody for 1 h in blocking buffer, washed in PBS, and incubated with the secondary antibody for 60 min in blocking buffer. After a final wash in PBS, coverslips, were placed onto a slide and covered with a mounting medium. DAPI (Sigma, D9542) was used in a solution in PBS, following the manufacturer’s instructions, for 5 min to detect nuclei.

Images were taken using an Olympus FV1000/BX61 microscope with 100x, 60 × (1.4 NA) oil-immersion objectives. For primary antibodies, we used mouse anti-**4R-Tau** (Millipore, 05-804, 1:100); mouse anti-**total tau** (Santa Cruz, sc-390476, 1:100); anti **pTDP-43** (Cosmo Bio TIP-PTD-M01, 1:100, Jacksonville, FL, ); mouse monoclonal **TDP-43** (Cell Signaling, #3449, 1:100); **pSer** (Stressmarq, SPC-149); anti-GSK3β (Cell Signaling, D85E12, 1:100).

Alexa-555 and Alexa-488-conjugated secondary antibodies, as well as DAPI and eGFP, were excited using Argon (lambda: 488 nm) and Helium-Neon (lambda: 543 nm), and with a 405 nm-UV lasers, and a transmission light detector. Optical sections for stacks were of 2 μm/steps. For comparisons, identical laser power and acquisition settings were used. For image processing, images were imported into ImageJ software (NIH).

### Cytoplasmic-to-nuclear ratio determination

To assess cytoplasmic-to-nuclear fluorescence intensity ratios, images were analyzed using ImageJ. The nuclear region of interest (ROI) was manually selected based on the DAPI channel. The cytoplasmic ROI, encompassing the entire cell including the nucleus, was delineated using the brightfield (light microscopy) channel. *Raw Integrated Density* values for both nuclear and total cell ROIs were measured after background subtraction. The cytoplasmic intensity was then calculated by subtracting the nuclear ROI signal from the total cell signal. The cytoplasmic-to-nuclear ratio was then calculated.

All materials were purchased from Sigma unless stated otherwise.

### Representative clinical samples

Brain samples were obtained from the Mayo Clinic neuropathological database in Rochester, Minnesota, from patients enrolled in a National Institutes of Health (NIH)-funded study conducted by the Neurodegenerative Research Group (Principal Investigator: K.J.). The Mayo Clinic Institutional Review Board (IRB) approved the study, and all patients or proxies consented to the research study. (The proxies provided consent for patients when needed.) The study followed the ethical standards of the Committee on Human Experimentation at Mayo Clinic by the Helsinki Declaration of 1975. Clinical trial number: not applicable.

For the purpose of this illustrative dataset, four representative patients were selected. All had undergone brain autopsy at the Mayo Clinic between November 15, 2019, and November 10, 2022. All four patients presented clinically with symptoms consistent with frontotemporal lobar degeneration (FTLD) and had histopathological confirmation of corticobasal degeneration (CBD). The first two cases exhibited neuropathological features of CBD. Case 1 was a 74-year-old man with a history of bradyphrenia and language impairment with striking anomia without loss of word meaning. Case 2 was a 75-year-old woman with behavioral changes and executive dysfunction consistent with behavioral variant frontotemporal dementia (bvFTD) with a medical history of dementia and type II diabetes mellitus (T2DM), confirmed by multiple point-of-care glucose tests. The remaining two cases were diagnosed neuropathologically with CBD and Limbic-Predominant Age-Related TDP-43 Encephalopathy Neuropathologic Changes (LATE NC), characterized by co-expression of abnormally phosphorylated truncated 4-repeat tau and TDP-43 isoforms. Case 3 was a 73-year-old woman with progressive behavioral changes, ideomotor apraxia and parkinsonism consistent with symmetric corticobasal syndrome. Case 4 was a 67-year-old woman with a three-year history of cognitive decline and behavioral changes consistent with bvFTD, and a history of elevated glucose levels in both outpatient and inpatient settings.

### Neuropathology- immuno-histochemistry

All patients underwent standardized neuropathological evaluation as previously described [24, 25]. Diagnoses were made by a board-certified neuropathologist (R.R.R.). Samples were selected to stain two brain regions: the hippocampus (HC) and the superior middle frontal gyrus (SMFG). Antibodies used as described previously (see *Detection of proteins by immunofluorescence).* Insulin DAB stainings were performed with a mouse monoclonal anti-insulin antibody (Santa Cruz, 2D11-H5, sc-8033, 1:2000). Hematoxylin was used as a counterstain. Imaging was performed using a standard digital slide scanner (Grundium Ocus 40).

### Data analysis and figure preparation

Data were analyzed by Student’s t-test or ANOVA followed by Tukey post-hoc tests, and plotted using Graph Pad Prism version 8.01 (Graph Pad Software Inc., La Jolla, San Jose, CA, USA). Images were designed using Adobe Illustrator CC version 10 software.

## Results

1. **Selective induction of 4R-tau expression and TDP-43 redistribution by insulin**.
  Mouse models used to study tau splicing frequently rely on transgenic animals expressing human tau, due to species-specific differences in isoform expression. In mice, 3R-tau predominates during embryonic development, while expression shifts toward 4R-tau postnatally, as illustrated in **Supplementary Fig. 1A**,** B**. Nonetheless, we employed wild-type C57 animals to examine whether endogenous 4R-tau expression could be induced at the embryonic stage using primary cortical cultures.
  As shown in Fig. 1A and B, insulin treatment was able to induce the expression of the 4R-tau isoform, which was undetectable in control cells by either western blot or immunofluorescence.The insulin-induced increase in 4R-tau was abolished by cycloheximide pre-treatment, indicating dependence on new protein synthesis (Supplementary Fig. 1C). Together with the observed insulin-driven TDP-43 phosphorylation, this suggests that insulin may activate a coordinated translational program leading to altered tau isoform expression.Insulin significantly increased 4R tau levels, suggesting that metabolic signaling may influence tau splicing even in developmentally immature neurons.
  Given previous findings by Gu et al. [10] on the role of TDP-43 in tau splicing and mRNA stability, we next asked whether insulin affects TDP-43 localization. In untreated cells, TDP-43 was primarily nuclear, with approximately 70% of the total signal localized to the nucleus. Insulin treatment altered this distribution, increasing the cytoplasmic fraction to 54% and reducing the nuclear fraction to 46% (Fig. 1C, D).
  To better visualize these changes, we turned to HEK293 cells overexpressing mCherry-tagged TDP-43. The mCherry-TDP-43 fusion construct was validated against antibodies for both TDP-43 and mCherry (**Supplementary Fig. 1C**), confirming consistent subcellular localization and signal specificity. As shown in Fig. 1D, insulin promoted a marked redistribution of TDP-43 toward the cytoplasm, with the cytoplasmic-to-nuclear signal ratio increasing from 0.007 ± 0.001 in control cells to 1.3 ± 0.1 in insulin-treated cells.
  **Fig. 1:**
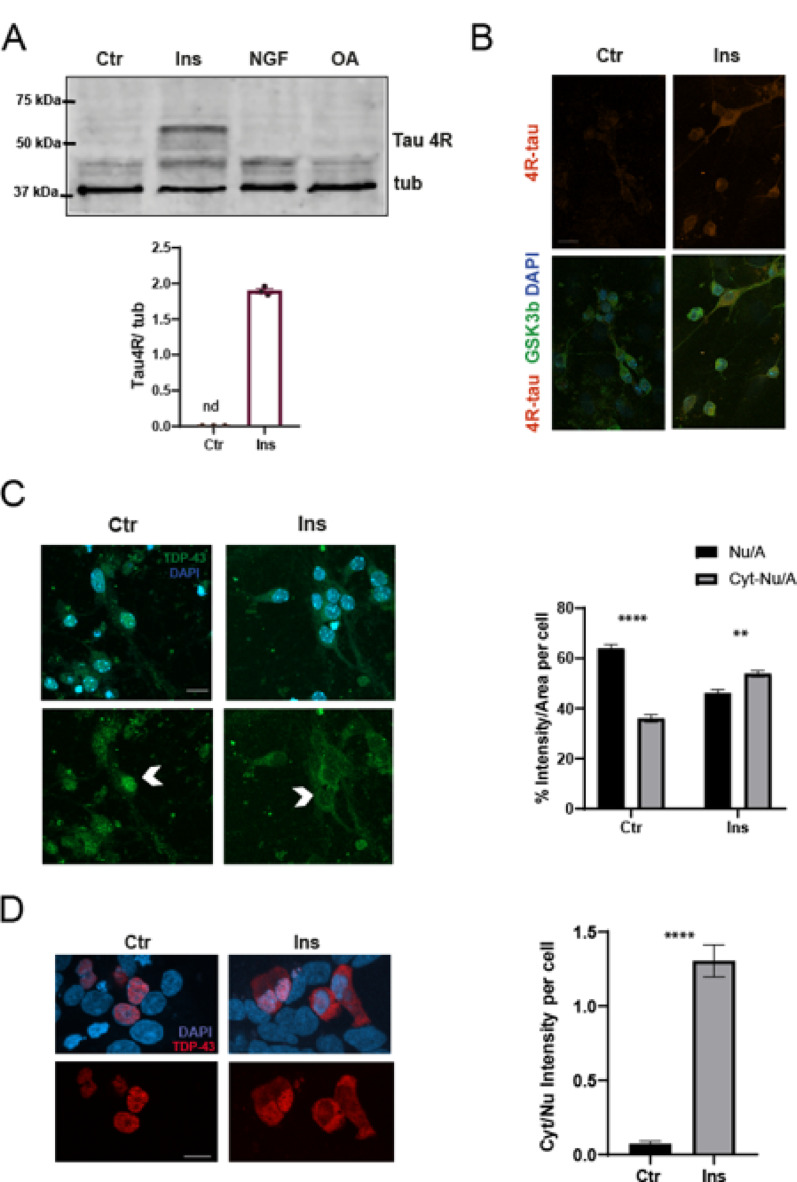
Insulin induces 4R-tau expression in primary neurons and promotes TDP-43 redistribution in neurons and HEK cells. **A** Representative Western blot (WB) of lysates from primary mouse cortical neurons treated with insulin (Ins), nerve growth factor (NGF) to induce CKII activity [26], okadaic acid (OA), or left untreated (Ctr), probed with an antibody against 4R-tau. A clear 4R-tau signal is detected exclusively in insulin-treated samples. The same membrane was subsequently incubated with a tubulin (tub) antibody, and both 4R-tau and tubulin signals are shown. Immunofluorescence images (right) of Ctr and Ins-treated neurons stained for 4R-tau (red), GSK3β (green), and DAPI (blue) confirm the presence of 4R-tau only in Ins-treated cells. **B** Quantification of 4R-tau levels normalized to tubulin from three independent experiments. Data are shown as mean ± SEM; nd, not detected. **C** Immunofluorescence images of Ctr and Ins-treated neurons stained for TDP-43 (green) and counterstained with DAPI. In Ctr cells, TDP-43 is predominantly nuclear, whereas insulin treatment leads to marked nuclear depletion and increased cytoplasmic accumulation. Quantification of TDP-43 intensity per area in nuclear and cytoplasmic compartments (soma excluding nuclei) is shown as a percentage; data are presented as mean ± SEM (*n* = 23–26 cells). Statistical analysis was performed using paired Student’s t-test (**p < 0.01; ****p < 0.0001). **D** Immunofluorescence images of HEK cells transfected with TDP-43, either untreated (Ctr) or treated with Ins, and counterstained with DAPI. Quantification of the cytoplasmic-to-nuclear signal ratio (Cyt/Nuc) is shown. A redistribution pattern similar to that observed in neurons is evident, with increased cytoplasmic localization due to insulin treatment. Statistical analysis was performed using paired Student’s t-test ****p < 0.0001). Scale bars, 10 μm. Given differences in signal intensity and morphology between primary neurons and HEK293 cells, TDP-43 redistribution was quantified using two distinct approaches: in neurons, the percentage of nuclear vs. cytoplasmic TDP-43 signal intensity per area was calculated, whereas in HEK cells, the cytoplasmic-to-nuclear (Cyt/Nuc) ratio was used to better reflect the redistribution pattern under overexpression conditions
2. ** Insulin leads to post-translational modifications in TDP-43**.
  To investigate whether insulin induces post-translational modifications in TDP-43, we analyzed mCherry-TDP-43–expressing HEK cells. As shown in Fig. 2A, insulin treatment resulted in a clear electrophoretic mobility shift compared to control cells. This shift was abolished when lysates were prepared without phosphatase inhibitors, suggesting a phosphorylation-dependent mechanism. A similar shift was also observed in insulin-treated primary cortical neurons (Fig. 2A, right). Supporting the involvement of phosphorylation, an increase in phospho-serine signal was detected at the molecular weight corresponding to TDP-43 in insulin-treated neurons (Fig. 2B). We further assessed phosphorylation at Ser409/410, a site associated with pathological TDP-43 aggregation. This phosphorylation was detected only in okadaic acid–treated neurons as expected [27], but not in insulin-treated cells (Fig. 2C), indicating that insulin promotes a distinct, non-pathological phosphorylation pattern.
  **Fig. 2:**
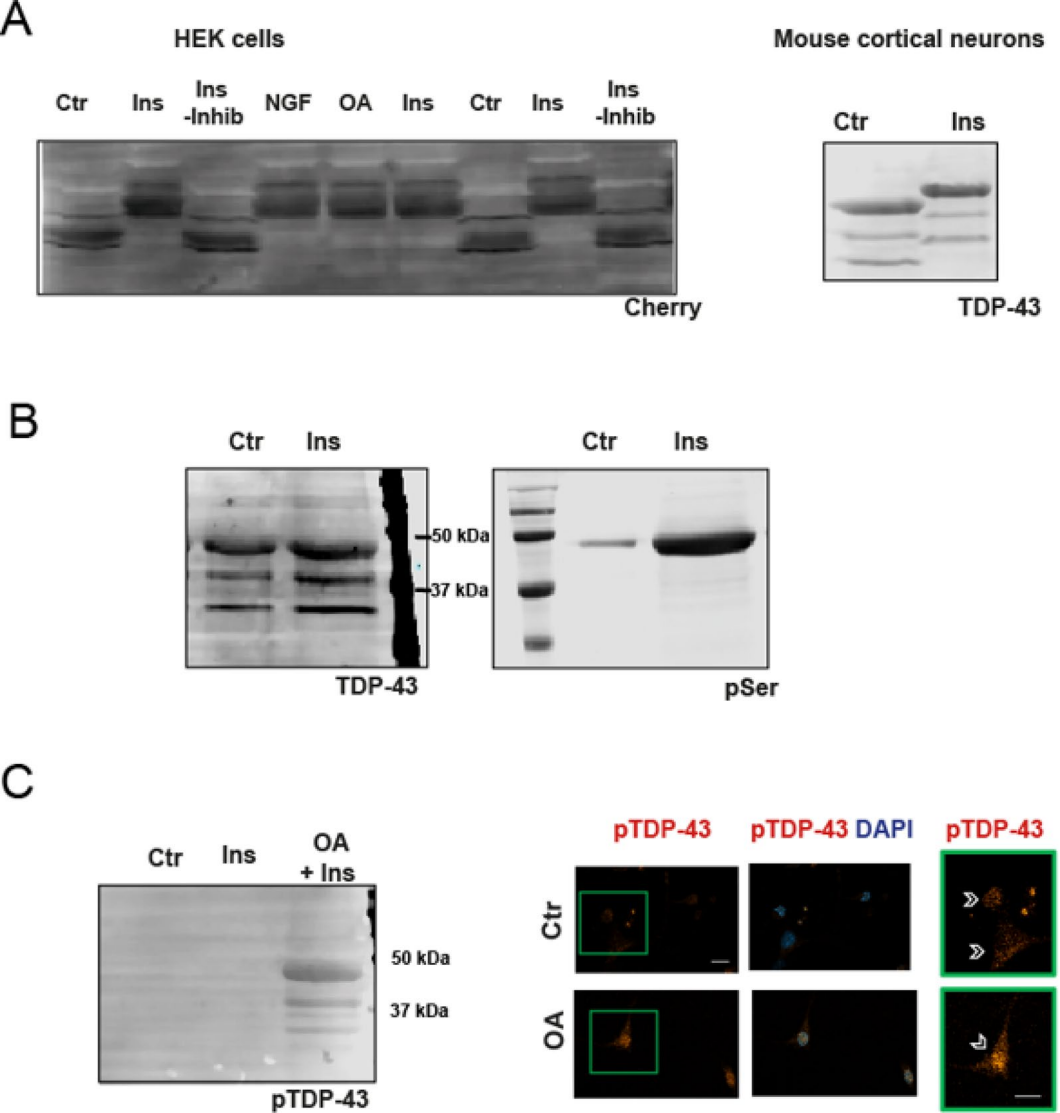
Figure 2 Insulin promotes electrophoretic changes in TDP-43 linked to post-translational modifications. **A** Representative Western blot (WB) showing electrophoretic mobility of mCherry-TDP-43 in lysates from HEK293 cells transfected with mCherry-TDP-43 and treated with insulin (Ins), nerve growth factor (NGF), okadaic acid (OA), or left untreated (Ctr). A distinct band shift indicating reduced electrophoretic mobility is observed in insulin-treated samples. This shift is abolished when phosphatase inhibitors are omitted during lysis (Ins – inhib), suggesting a phosphorylation-dependent modification. A similar shift is observed in cortical neurons treated with insulin (right panel). Notice that “mobility gels” were run under modified conditions (See Material and Methods) that do not allow reliable marker migration. **B** Representative WB showing TDP-43 detection in control and insulin-treated primary neurons, alongside the same membrane probed with an anti-phospho-serine (pSer) antibody. The Ins-treated sample shows a stronger pSer signal at the same molecular weight as TDP-43, indicating increased serine phosphorylation upon insulin treatment. Notice that “mobility gels” in **A** and **B** were run under modified conditions (See Material and Methods) that do not allow reliable marker migration. **C** WB analysis using a phospho-specific antibody against TDP-43 phosphorylated at Ser409/410 (pTDP-43) in neurons treated with Ctr, Ins, or OA. A specific pTDP-43 band is detected only in OA-treated cells, but not in control or insulin-treated neurons. Right: Immunofluorescence images showing nuclear accumulation of pTDP-43 (Ser409/410) in OA-treated neurons (arrowheads in enlarged inset) compared to Ctr and with no detectable signal in insulin-treated cells (not shown). Scale bar, 10 μm
3. ** 4R-tau induction is associated with CK1-dependent TDP-43 phosphorylation**.
  To investigate the involvement of kinases acting on TDP-43 in 4R-tau induction, we pretreated cortical neurons with kinase and phosphatase inhibitors prior to insulin stimulation. As shown in Fig. 3A, only D4476, a casein kinase 1 (CK1) inhibitor, attenuated insulin-induced 4R-tau expression, while DMAT (CK2 inhibitor) and okadaic acid (OA; PP1a/PP2a inhibitor—tested at two concentrations (1× and 10×), had no appreciable effect. Pretreatment with D4476 also abolished the insulin-induced electrophoretic mobility shift of TDP-43 (Fig. 3B), suggesting a CK1-dependent phosphorylation mechanism regulating both TDP-43 and 4R-tau.
  These results support the notion that insulin regulates tau splicing through phosphorylation-dependent changes in TDP-43, whereas other stimuli such as NGF or OA may induce distinct phosphorylation patterns not directly linked to 4R tau modulation.
  A time-course analysis (Fig. 3D) revealed a progressive reduction in 4R-tau levels with increasing D4476 pre-incubation durations (1, 2, and 4 h), culminating in complete loss of the 4R band after 4 h. Quantification confirmed this marked decrease in 4R-tau relative to tubulin. This inhibition was further confirmed in Supplementary Fig. 1B, where after 4 h of D4476 pretreatment, only a lower molecular weight band consistent with 3R-tau remained.
  Given that TDP-43 has been implicated in tau pathology, we evaluated whether TDP-43 overexpression could modulate tau levels independently of insulin signaling. As shown in Supplementary Fig. 2, co-expression of TDP-43 significantly reduced the levels of tau-441wt and other tau constructs, suggesting a broader regulatory role of TDP-43 on tau expression.
  **Fig. 3:**
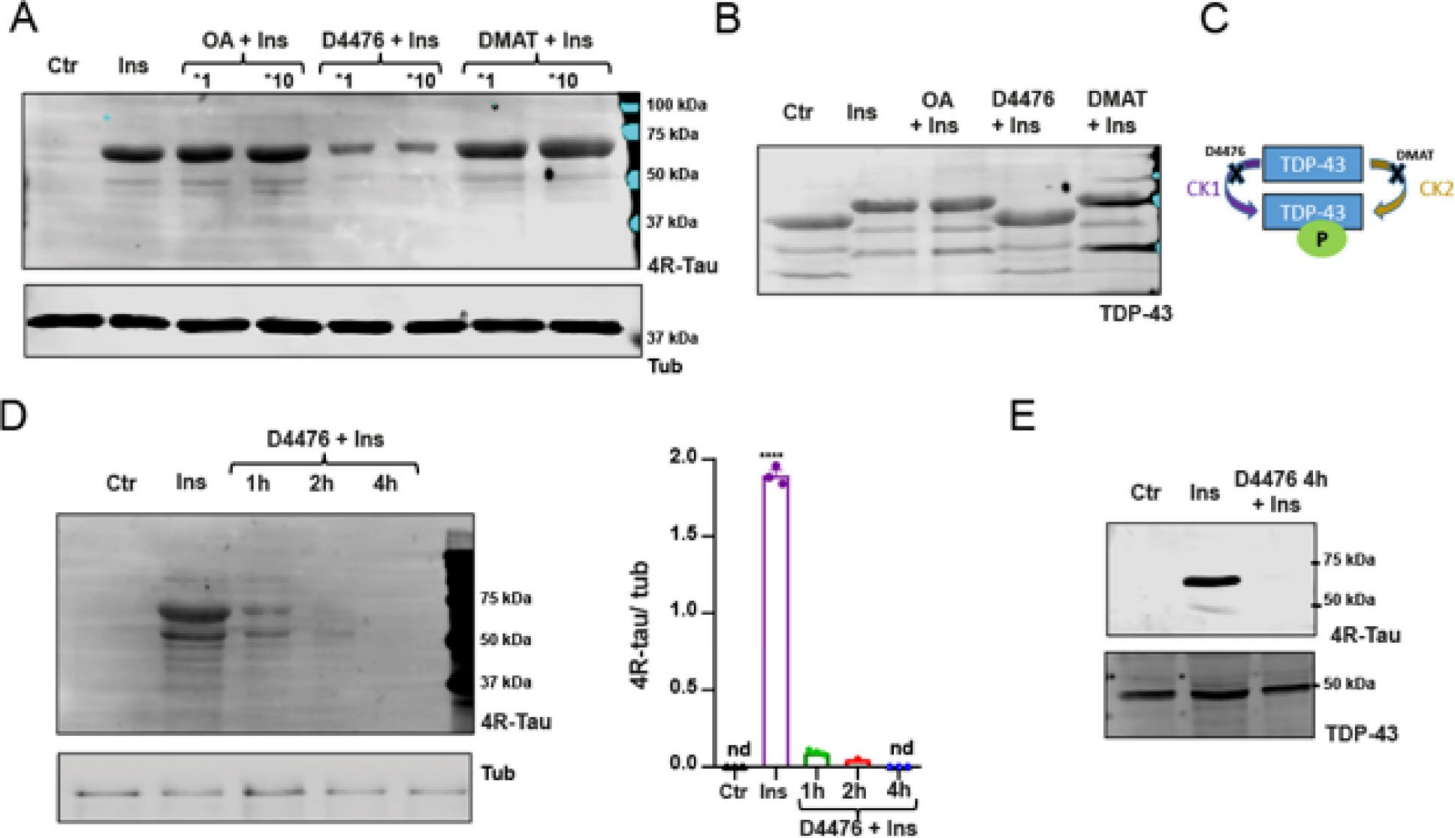
Inhibition of CK1 reduces insulin-induced 4R-tau expression and TDP-43 phosphorylation in cortical neurons. **A** Representative Western blot (WB) of cortical neuron lysates pretreated for 1 h with kinase and phosphatase inhibitors prior to insulin (Ins) stimulation. Inhibitors included D4476 (CK1 inhibitor), DMAT (CK2 inhibitor), and okadaic acid (OA; PP1a/PP2a inhibitor), each tested at two concentrations (×1 and ×10). Detection with a 4R-tau –specific antibody reveals a marked reduction in the insulin-induced 4R-tau signal only in D4476 + Ins-treated neurons, with both concentrations showing strong inhibition. D4476 at ×1 significantly reduces 4R tau induction. **B** WB showing electrophoretic mobility of TDP-43 under the same treatment conditions as in (A). The mobility shift observed in Ins- and OA-treated samples (consistent with phosphorylation) is absent in D4476 + Ins-treated neurons, supporting CK1 involvement in TDP-43 phosphorylation. **C** Schematic of CK inhibitors used: D4476 selectively inhibits CK1, and DMAT inhibits CK2, both kinases implicated in TDP-43 phosphorylation **D** Representative WB showing the effect of different pre-incubation times (1, 2, and 4 h) with D4476 (×1) prior to insulin stimulation. A time-dependent decrease in 4R-tau signal is observed, indicating progressive inhibition of insulin-induced expression. Right: Quantification of 4R-tau levels normalized to tubulin from three independent experiments. Insulin treatment significantly increases 4R-tau expression compared to control, and this induction is progressively inhibited by D4476 pretreatment (****p < 0.0001, one-way ANOVA followed by Tukey’s post hoc test). No 4R-tau signal was detected in untreated (Ctr) or in the 4 h D4476 + Ins condition (nd). **E** Representative membranes showing 4R-tau (upper panel) and TDP-43 (lower panel) expression in control (Ctr), Ins, and D4476 (4 h pre-incubation) + Ins conditions. While 4R-tau levels increase upon insulin stimulation and are reduced by D4476, TDP-43 levels remain unchanged across conditions
4. **Region-specific 4R-tau induction in cortex and hippocampus**.
  Given the regional vulnerability observed in diseases such as Alzheimer’s disease (AD) and frontotemporal lobar degeneration (FTLD), we also compared the extent of 4R-tau induction in cortical and hippocampal tissues. As shown in Fig. 4, mouse hippocampal neuronal cultures exposed to insulin induced 4R-tau protein expression. Additionally, a stronger induction was detected in insulin-treated hippocampal lysates compared to cortical ones, indicating possible region-specific sensitivity to insulin signaling (Fig. 4, B).
  **Fig. 4:**
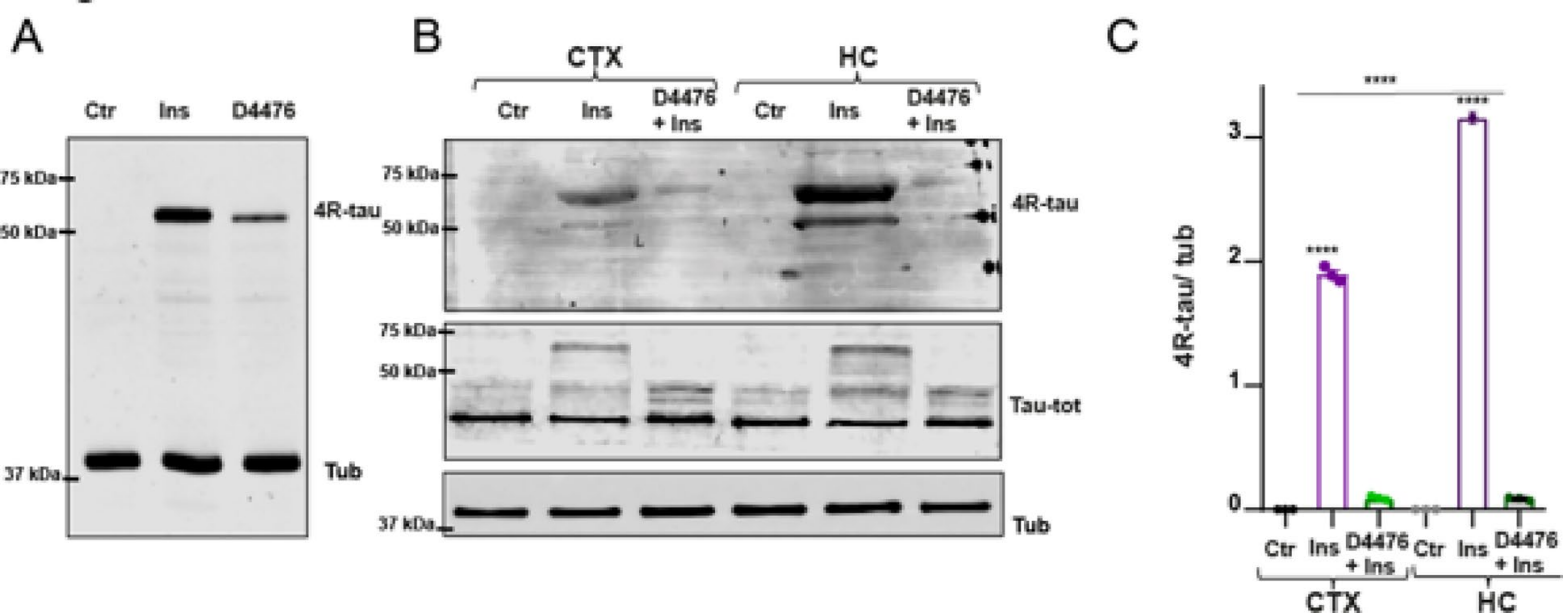
Induction of 4R-tau in Cortical and Hippocampal neurons. **A** Representative Western blot (WB) of hippocampal neuron lysates treated with insulin (Ins), D4476 (CK1 inhibitor) for 1 h prior to insulin stimulation, or left untreated (Ctr). Membranes were probed with a 4R-tau –specific antibody (upper panel) and tubulin (lower panel) as a loading control. **B** WB comparing 4R-tau expression in lysates from insulin-treated cortex (CTX) and hippocampus (HC), showing stronger induction in HC samples. Middle panel shows total tau detection using a pan-tau antibody, included to visualize tau bands in samples lacking detectable 4R tau. Lower panel shows tubulin as a loading control to confirm similar protein input across samples. **C** Quantification of 4R-tau normalized to tubulin (4R-tau /Tub) from three independent experiments. No signal was detected in untreated controls. Insulin significantly increased 4R-tau levels in both CTX and HC compared to their respective controls (****p < 0.0001). This induction was prevented by D4476 pretreatment (****p < 0.0001 vs. Ins). Additionally, insulin-induced 4R-tau expression was significantly higher in HC compared to CTX (****p < 0.0001). One-way ANOVA followed by Tukey’s post hoc test
5. ** Neuropathological observations of 4R-tau in CBD patients with metabolic comorbidities**.
  To complement our in vitro findings, we examined postmortem brain tissue from a small sample of neuropathologically confirmed corticobasal degeneration (CBD) patients (*n* = 4). Corticobasal degeneration is a primary tauopathy characterized by predominant accumulation of four-repeat tau (4R-tau). The selected cases included individuals with and without type II diabetes mellitus (T2DM).
  Immunostaining for 4R-tau was examined in the left hippocampal dentate gyrus (dg), subiculum (sb), and the grey (gm) and white matter (wm) of the superior and middle frontal gyrus (SFMG). Compared to a CBD patient without hyperglycemia history (Case 1), increased 4R-tau immunoreactivity was qualitatively observed in the T2DM patient (Case 2). Similarly, the patient with combined CBD and LATE NC (Case 3) showed greater 4R-tau burden relative to Case 1, with the highest tau pathology detected in the patient presenting both LATE NC and T2DM (Case 4) (Fig. 5). As shown in the images, TDP-43–positive aggregates are distributed across multiple cellular compartments, including nuclear inclusions, neuronal cytoplasmic inclusions, and dystrophic neurites. Granulovacuolar degeneration was also occasionally observed. No clear predominance of any specific inclusion type was noted.
  Phosphorylated TDP-43 (pTDP-43) staining showed no appreciable differences across cases. These results are in line with our in vitro findings, where insulin increased 4R-tau expression in neurons, without corresponding changes in pTDP-43 signal detected at that specific site (Ser409/410).
  **Fig. 5:**
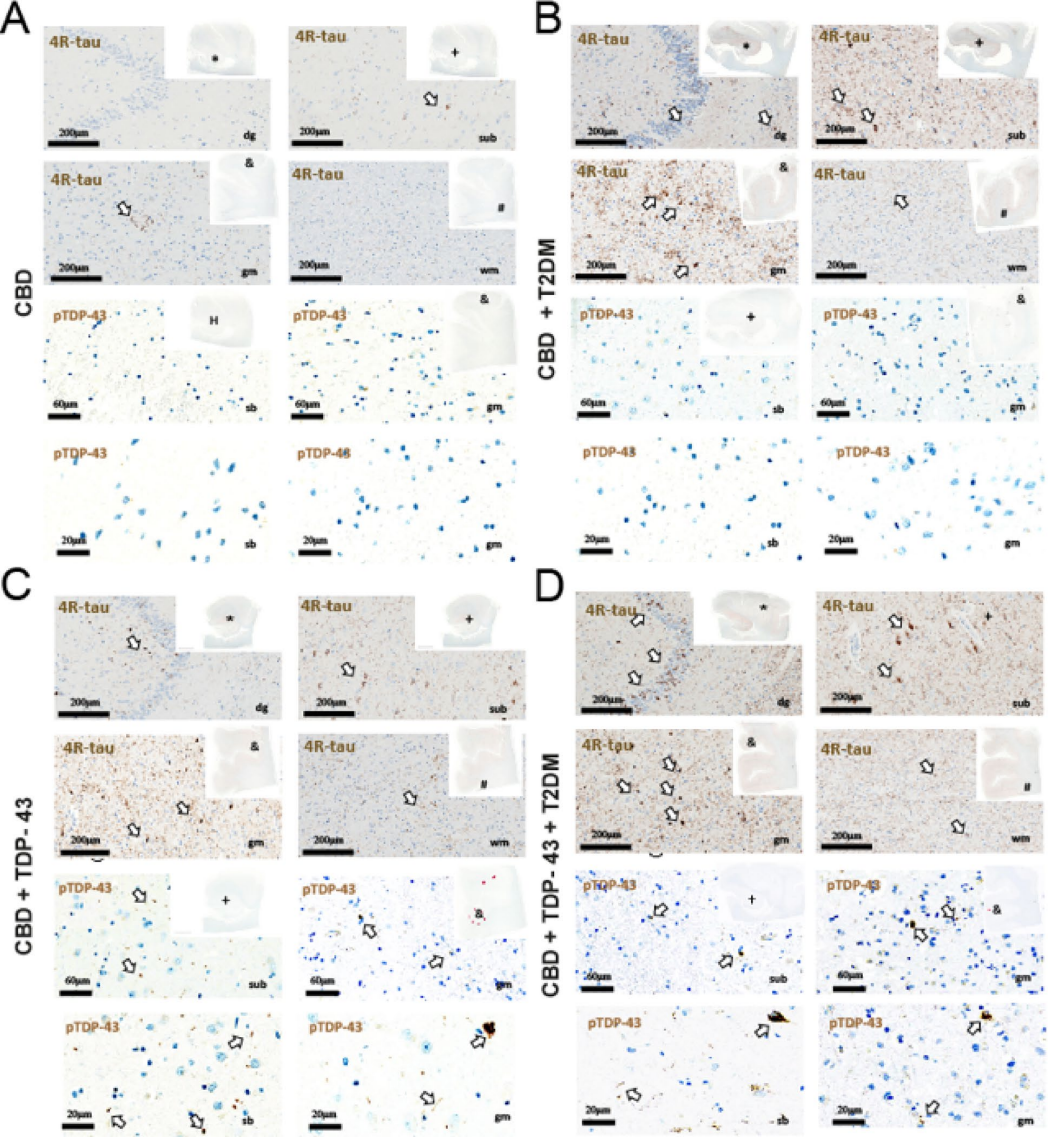
4R-tau and TDP-43 immunostaining in CBD patients with or without metabolic comorbidities Histopathological analysis of 4R-tau and phosphorylated TDP-43 (Ser409/410) in brain sections from a representative subset of four neuropathologically confirmed corticobasal degeneration (CBD) patients. Insets in each panel show topographic brain views indicating the anatomical location of the sampled regions. **A** Sections from a CBD patient without known metabolic comorbidities (Case 1), including the left hippocampal dentate gyrus (dg, upper right) and subiculum (sb, lower right), as well as the superior and middle frontal gyrus (SFMG), covering grey matter (gm) and white matter (wm). **B** 4R-tau stainings from a CBD patient with comorbid type II diabetes mellitus (T2DM) (Case 2), across the same brain regions. **C** Sections from a CBD patient with comorbid limbic-predominant age-related TDP-43 encephalopathy (LATE), showing TDP-43-positive pathology (Case 3). **D** Sections from a CBD patient with both LATE and a history of T2DM (Case 4). White arrows indicate 4R-tau –positive ballooned neurons and, in cases 3 and 4, TDP-43–positive inclusions. No TDP-43 immunoreactivity was observed in cases 1 and 2. Hematoxylin was used as a counterstain. Scale bars: overview panels = 200 μm; insets = 60 μm
  In parallel, we evaluated insulin immunoreactivity in adjacent brain regions from the same cases (**Supplementary Fig. 3**). While insulin staining was absent in the CBD case without T2DM (Case 1), a clear positive signal was detected in Case 2, who presented CBD and T2DM. Similarly, insulin staining was observed in Case 4 (CBD + TDP-43 + T2DM), but not in Case 3 (CBD + TDP-43). Although insulin was not directly detected in the hippocampus or cortical parenchyma where 4R-tau was assessed, these findings provide evidence for the presence of insulin within the CNS in patients with T2DM. Given that these same cases showed increased 4R-tau immunoreactivity in hippocampal and frontal regions, these data are consistent with our in vitro observations linking insulin exposure to increased 4R-tau expression, and support the hypothesis that insulin may modulate tau pathology in vivo.

## Discussion

We have analyzed the effect of insulin in neuronal cells and showed that this stimulus can induce the expression of 4R-tau isoform at an embryonic stage in which only the 3R isoform is typically expressed. This effect highlights the potential role of metabolic signaling in the regulation of tau splicing, a process of increasing interest in the context of neurodegenerative diseases. While physiological brain insulin levels are generally below 0.1 nM, and increase only modestly under hyperinsulinemic states, our higher in vitro concentration was chosen to elicit a maximal cellular response, with comparable but weaker effects also observed at near-physiological levels (Supplementary Fig. 1C). Previous studies have shown impaired insulin signaling results in PI3K-Akt and Wnt-β-catenin pathway decreased activity, and activation of GSK3β, thus promoting tau phosphorylation. As such, the insulin/IGF pathway dysfunctions are linked to cortical atrophy, loss of synaptic plasticity, white matter myelin/oligodendrocyte degeneration, astrocyte and microglia neuroinflammation and oxidative stress, deficits in energy metabolism, mitochondrial dysfunction, and microvascular disease [28, 29]. In Alzheimer’s disease (AD) and cognitive decline, several groups have reported reduced insulin levels in the brain [1, 13, 14, 30]. According to our findings, such deficits could impair the induction of 4R-tau, thereby disrupting the physiological 3R/4R tau balance. This may help explain reports detecting an increase detection of 3R-tau in tau inclusions from AD patients, as well as in other disorders associated with a higher 3R/4R ratio.

Additionally, we observed that insulin treatment affected TDP-43 post-translational modifications and subcellular localization, correlating with the shift in tau isoform expression. This in fact relates to a potential interplay with tau and TDP-43 [31].TDP-43, a known regulator of mRNA splicing, which is a known key component in RNA splicing due to its ability to bind both introns and exons [12], has been associated with both canonical splicing regulation, and the suppression of cryptic exon inclusion [12, 32, 33]. Gu et al. analyzed TDP-43’s role in tau exon 10 splicing and found that phosphorylation at Ser403/404 and Ser409/410, which promotes TDP-43 mislocalization to the cytoplasm, correlated with reduced exon 10 inclusion — resulting in decreased 4R-tau isoform expression [34]. Their studies, conducted in HEK293, N2a, and SH-SY5Y cells, support a model in which nuclear TDP-43 is required for proper exon 10 inclusion. Our findings partially align: while we also observed insulin-induced TDP-43 redistribution and phosphorylation, we did not detect a consistent increase in Ser409/410 phosphorylation accompanying 4R-tau induction, nor a decrease in 4R-tau. On the contrary, we found that 4R-tau expression increased despite the absence of detectable phosphorylation at Ser409/410 in vitro (Fig. 2), and no significant differences in this site were observed among human brain samples (Fig. 5), suggesting the involvement of alternative phosphorylation sites or additional regulatory mechanisms. Gu et al. had linked TDP-43 mislocalization and phosphorylation at Ser403/404 and Ser409/410 to decreased exon 10 inclusion and reduced 4R-tau levels. Thus, while their findings implicate phosphorylation at these sites in canonical splicing repression, our data suggest that phosphorylation at other sites may instead be associated with 4R-tau upregulation. Although cryptic exons have not been reported in the *MAPT* gene to date, the possibility remains worth exploring given the complexity of tau splicing regulation and the pathological importance of maintaining a proper 3R/4R isoform balance. In addition, other RNA-binding proteins such as FUS and SFPQ have been shown to participate in the regulation of tau exon 10 splicing and overall mRNA metabolism [35]. While our present work focused on TDP-43, the potential involvement of these factors in insulin-induced alterations of tau splicing represents an important question for future studies.

Phosphorylation-dependent cytosolic redistribution of TDP‑43 has been linked to CK1 activity [34], and our data supports this model. The CK1 inhibitor D4476 not only reversed insulin-induced TDP‑43 mobility shifts, but also progressively inhibited 4R-tau expression in a time-dependent fashion—1 h of preincubation partially reduced induction, while 4 h eliminated it. In a different model, Rena et al. demonstrated that a 60-minute preincubation of D4476 efficiently blocked CK1 phosphorylation of FOXO1a in H4IIE hepatoma cells [36]. In contrast, the complete inhibition observed at 4 h in our system may reflect a more complex mechanism, in which CK1-mediated phosphorylation of TDP-43 leads to its cytosolic redistribution, thereby altering tau pre-mRNA splicing and promoting the expression of the 4R-tau isoform. This multistep process likely requires sustained CK1 inhibition to fully block the downstream consequences of TDP‑43 mislocalization. Further studies are needed to elucidate the precise molecular events linking CK1, TDP‑43 phosphorylation, and tau isoform expression.

It is also worth noting that TDP-43 binds UG-rich sequences, which are known to regulate not only alternative splicing in the nucleus but also mRNA stability, particularly when located in the 3′ untranslated region (3′UTR). As reported by Gu et al. [34], TDP-43 mislocalization to the cytoplasm, as observed in our system, contributes to tau mRNA destabilization. Although our tau expression constructs were not specifically designed to assess mRNA stability, we observed reduced reporter expression in the presence of exogenous TDP-43 (Supplementary Fig. 2). While this system is not optimal for dissecting mRNA turnover, the observation opens the possibility that TDP-43 may regulate tau at multiple levels — both via splicing and post-transcriptionally.

Discrepancies between our findings and those of Gu et al. may stem from differences in the cellular systems used (primary mouse neurons versus immortalized cell lines) and the presence of insulin, which introduces additional regulatory effects. The use of primary neuronal cultures also allowed us to examine regional specificity, revealing a more pronounced induction of the 4R-tau isoform in the hippocampus compared to cortical regions. This regional sensitivity may reflect inherent differences in vulnerability, with the hippocampus being one of the regions of selective initiation in AD [37]. Notably, TDP-43 pathology has been associated with selective atrophy in hippocampal subfields such as CA1 and the subiculum, not only in FTLD but also in primary age-related tauopathy (PART), suggesting a shared regional susceptibility across conditions [38, 39]. In our study, increased 4R-tau immunoreactivity was observed in postmortem brain tissue from CBD patients with comorbid T2DM and/or TDP-43 pathology.

While hyperinsulinemic states are commonly associated with medical conditions such as type 2 diabetes mellitus (T2DM) [40], their effects on the central nervous system remain an area of active investigation. It has been reported that insulin resistance in both Alzheimer’s disease (AD) and T2DM can lead to hyperinsulinemia by saturating insulin-degrading biochemical pathways through impaired insulin signaling. In addition emerging evidence supports a strong association between hyperinsulinemia, cognitive decline [41], and AD pathology [15, 42, 43]. However, our understanding of insulin’s role in frontotemporal lobar degeneration (FTLD) and its underlying biochemical mechanisms remains limited. Nonetheless, alterations of glucose metabolism and increased insulin levels in blood have been seen in patients with behavioral variant of frontal temporal degeneration [44, 45]. Thus, our preliminary neuropathological assessment across FTLD-tau subtypes (with co-occurring TDP-43 pathologies) may help initiate a broader discussion on the potential link between insulin dysfunction and the increased presence of 4-repeat tau isoforms in these cases, a hypothesis further supported by the detection of insulin in CNS tissue from CBD patients with T2DM. While these results are based on a limited sample and remain descriptive, they provide preliminary support for an association between metabolic alterations and 4R-tau pathology.

In agreement, our in vitro findings show that insulin stimulation promotes 4R-tau expression in cortical and hippocampal neurons.

The overlap in brain regions affected by both TDP-43 and 4R-tau pathology—together with their apparent modulation by metabolic states—suggests that insulin may act as a physiological regulator of tau isoform expression in disease-relevant contexts.

Taken together, our results reveal that neuronal splicing machinery can be modulated by physiological cues such as insulin to induce isoforms not normally expressed at this developmental stage. Given the involvement of insulin signaling in neurodegeneration and the reported cognitive benefits of anti-diabetic treatments [46], the regulation of tau isoform expression by insulin warrants further investigation.

### Limitations

This study has several limitations that should be acknowledged.

First, the in vitro experiments were conducted in embryonic mouse cortical neurons, which may not fully recapitulate the molecular environment or pathological processes occurring in aged human neurons.

Second, the analysis of human brain tissue was descriptive and based on a limited number of cases, precluding a comprehensive evaluation of interindividual variability or disease-specific patterns. A larger number of samples would be helpful in the future to further strengthen these findings. Along this line, since CK1 expression in the brain tissue of CBD cases, particularly in the hippocampus, has been reported to accumulate in granulovacuolar degeneration (GVD) [47], detecting CK1 in samples would also provide important additional information regarding this mechanism.

Finally, although our data support a link between TDP-43 phosphorylation and 4R-tau upregulation, direct causal evidence remains to be established. Nevertheless, the converging in vitro and human findings presented here provide a strong basis for this proposed connection.

Future studies using humanized models and larger case cohorts will be required to validate and further expand these observations.

## Supplementary Information

Below is the link to the electronic supplementary material.

Supplementary Material 1

Supplementary Material 2

## Data Availability

All data generated or analyzed during this study are included in this published article and its supplementary information files. Additional details are available from the corresponding author upon reasonable request.

## Abbreviations

bvFTD: Behavioral variant frontotemporal dementia
BBB: Blood–brain barrier
CSF: Cerebrospinal fluid
CP: Choroid plexus
Ctr: Control
CTX: Cortex
CBD: Corticobasal degeneration
Cyt/Nuc: Cytoplasmic-to-nuclear
dg: Dentate gyrus
4R-tau: Four-repeat tau
FTLD-U: Frontotemporal Lobal Degeneration with Ubiquitin-positive inclusions
FTLD: Frontotemporal lobar degeneration
HC: Hippocampus
Ins: Insulin
LATE NC: Limbic-Predominant Age-Related TDP-43 Encephalopathy Neuropathologic Changes
MW: Molecular weight
ND: Not detected
NGF: Nerve growth factor
NDs: Neurodegenerative diseases
NFTs: Neurofibrillary tangles
OA: Okadaic acid
PART: Primary age-related tauopathy
Sb: Subiculum
SMFG: Superior middle frontal gyrus
TDP-43TAR: DNA binding protein 43
3R-tau: Three-repeat tau
Tub: Tubulin
T2DM: Type 2 diabetes mellitus
WB: Western blotting
wm: White matter
