# Supplementary material for: 4R-tau isoform induction via TDP-43 in neurons in response to insulin: converging signaling pathways with implications for neurodegenerative disease
Source: Acta Neuropathol Commun. 2025 Dec 24;13:258. doi: 10.1186/s40478-025-02174-x (PMC12729092; doi:10.1186/s40478-025-02174-x)
Supplement: Supplementary file 1 — Supplementary Material 1 [file 40478_2025_2174_MOESM1_ESM.pdf]

Figure 1

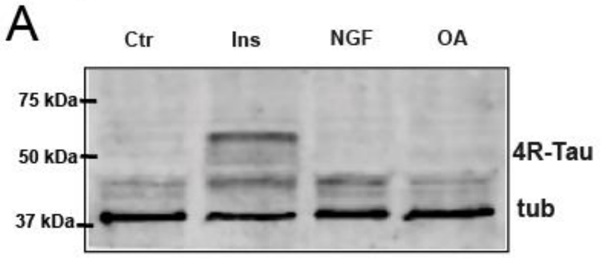

Figure 2

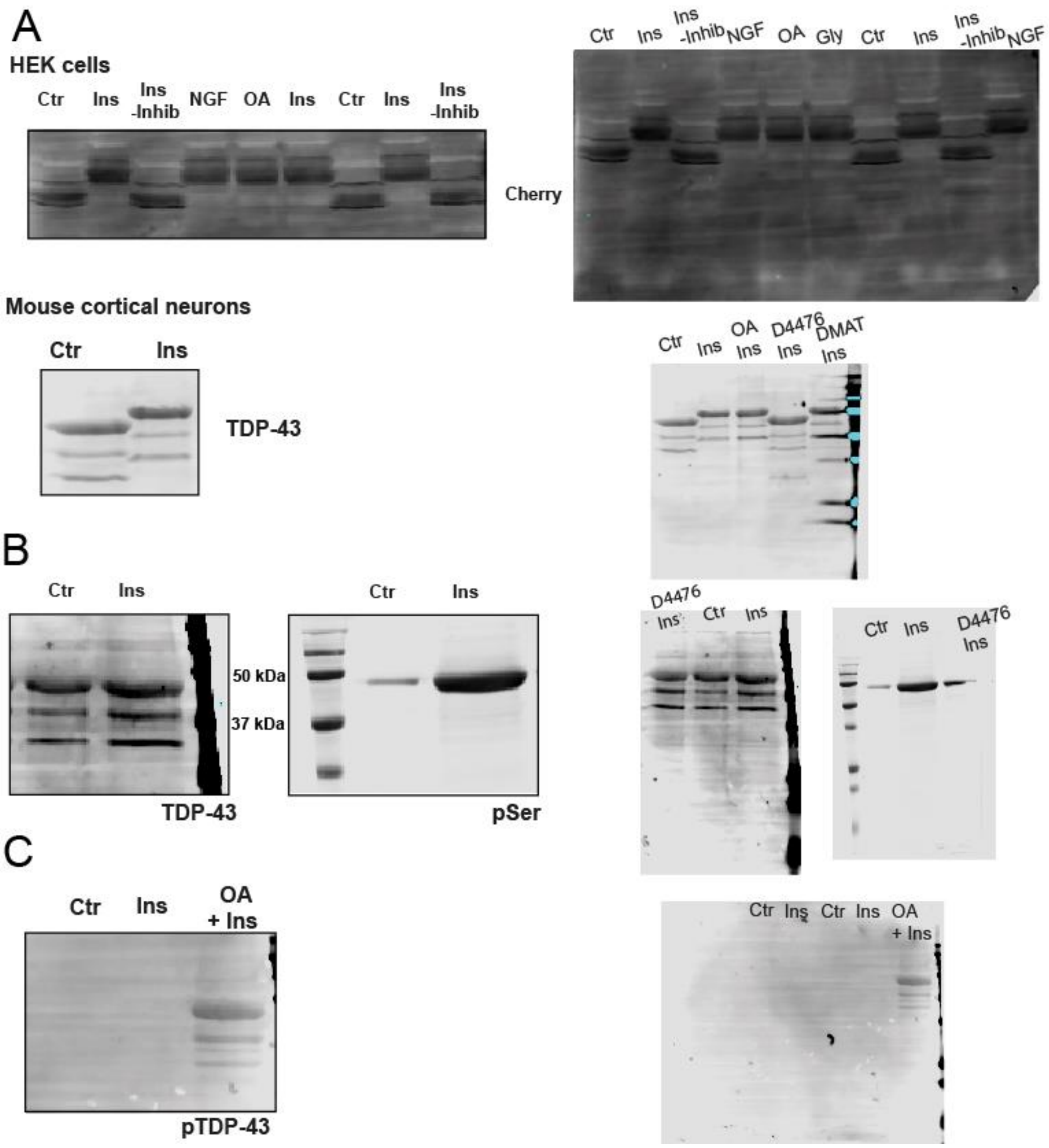

Figure 3

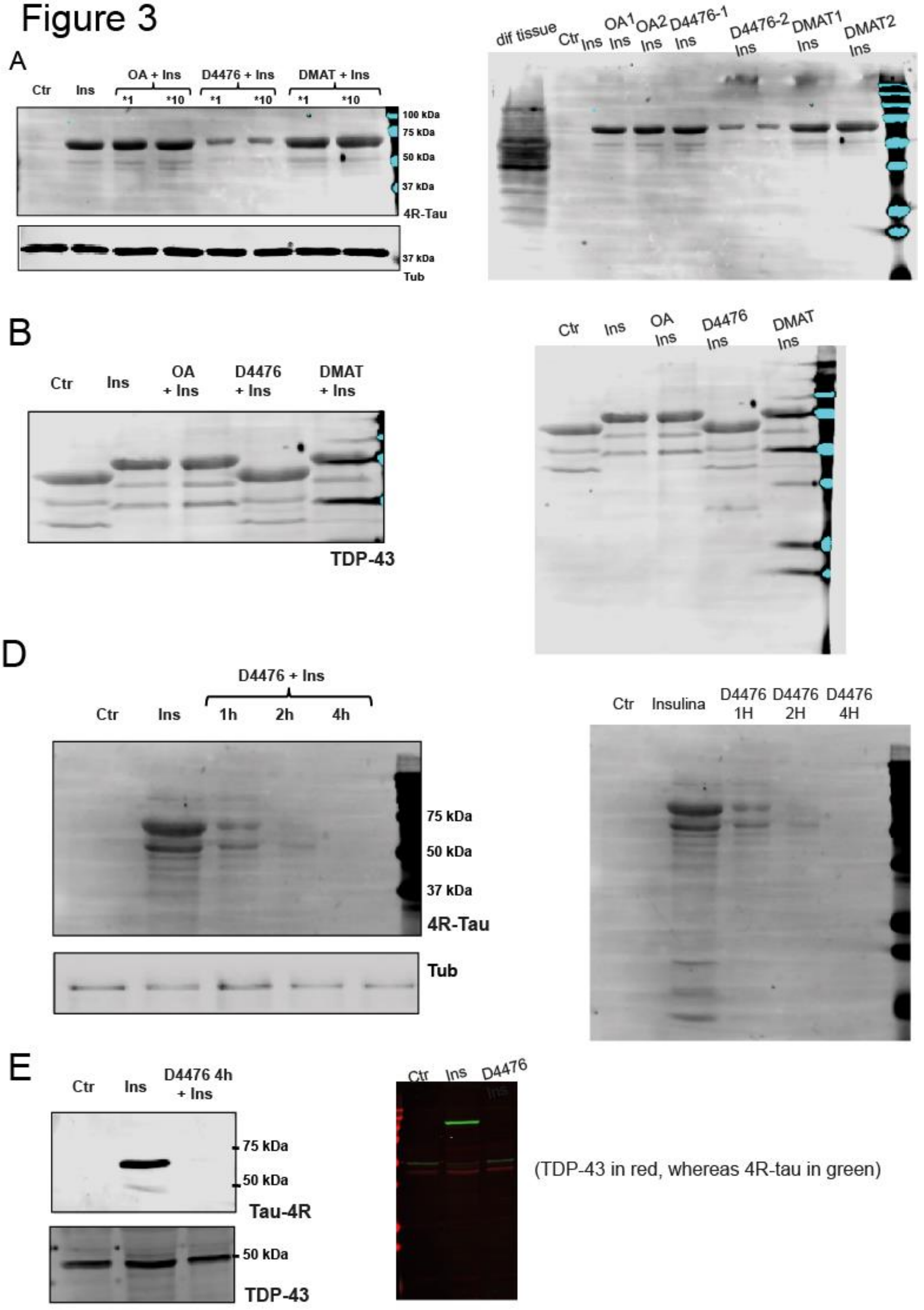

A
Ctr
Ins
OA + Ins
D4476 + Ins
DMAT + Ins
*1
*10
100 kDa
75 kDa
50 kDa
37 kDa
4R-Tau
37 kDa
Tub
dif tissue
Ctr
Ins
OA1 Ins
OA2 Ins
D4476-1 Ins
D4476-2 Ins
DMAT1 Ins
DMAT2 Ins
B
Ctr
Ins
OA + Ins
D4476 + Ins
DMAT + Ins
TDP-43
Ctr
Ins
OA Ins
D4476 Ins
DMAT Ins
D
D4476 + Ins
Ctr
Ins
1h
2h
4h
75 kDa
50 kDa
37 kDa
4R-Tau
Tub
Ctr
Insulina
D4476 1H
D4476 2H
D4476 4H
E
Ctr
Ins
D4476 4h + Ins
75 kDa
50 kDa
Tau-4R
50 kDa
TDP-43
Ctr
Ins
D4476 Ins
(TDP-43 in red, whereas 4R-tau in green)

Figure 4

A

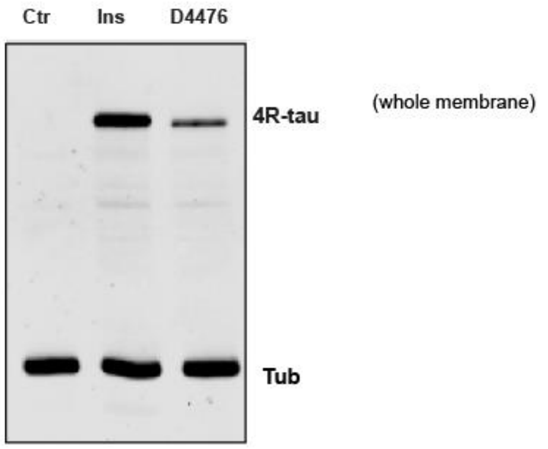
Ctr
Ins
D4476
4R-tau
(whole membrane)
Tub

B

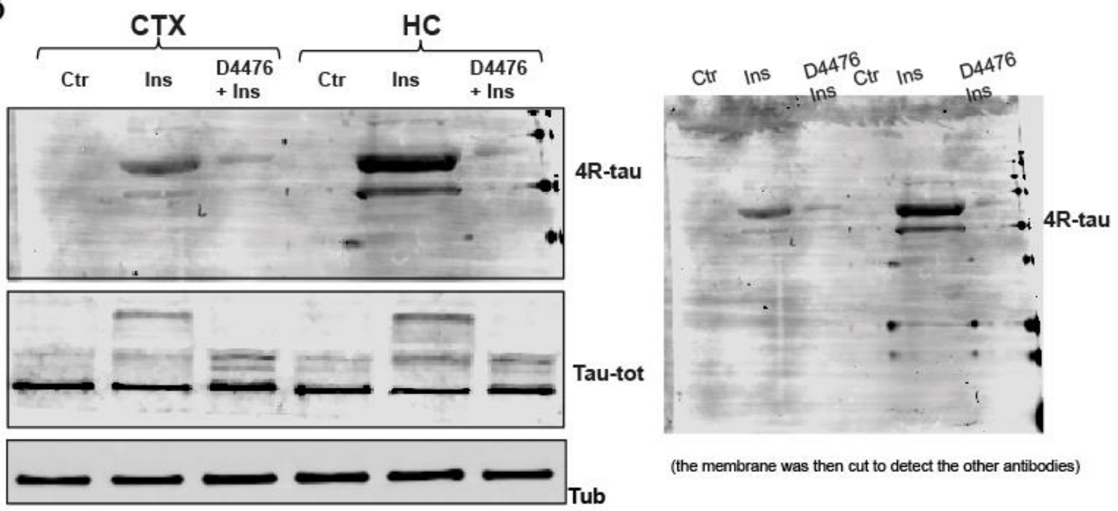
CTX
HC
Ctr
Ins
D4476 + Ins
Ctr
Ins
D4476 + Ins
4R-tau
Tau-tot
Tub
Ctr
Ins
D4476 Ins
Ctr
Ins
D4476 Ins
4R-tau
(the membrane was then cut to detect the other antibodies)

Supplementary Figure 1

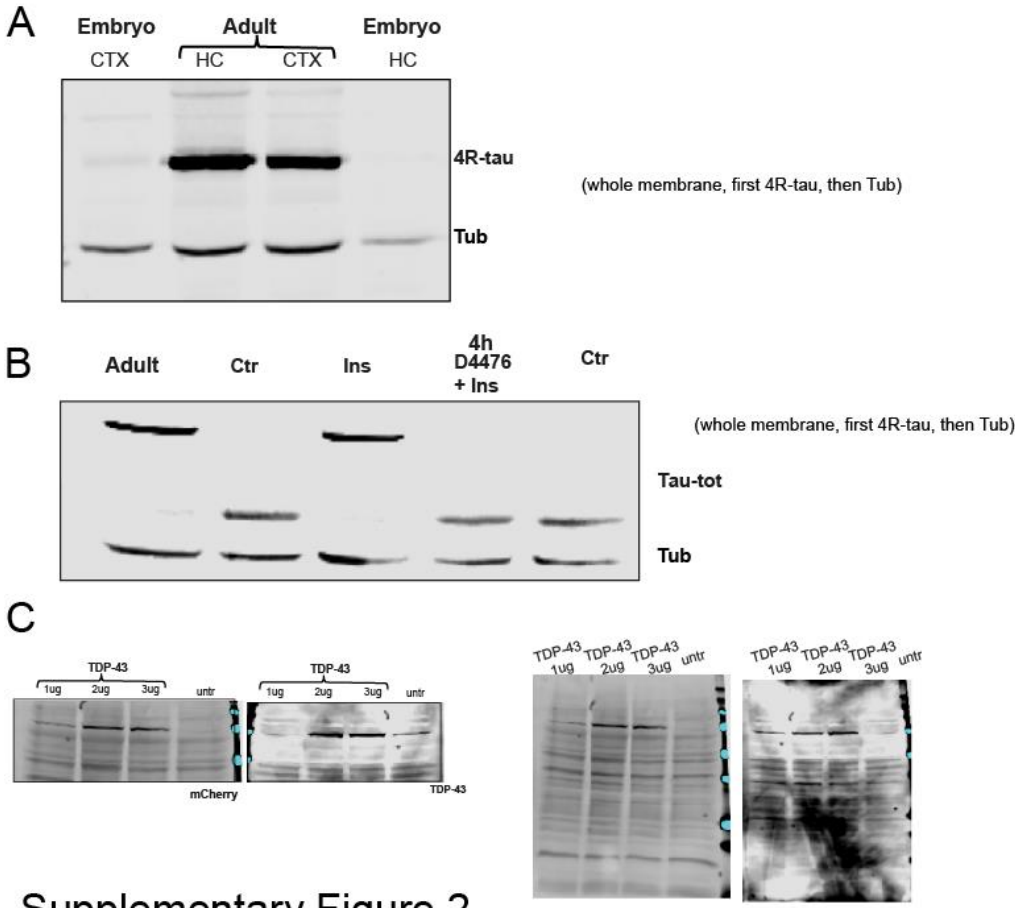

Supplementary Figure 2

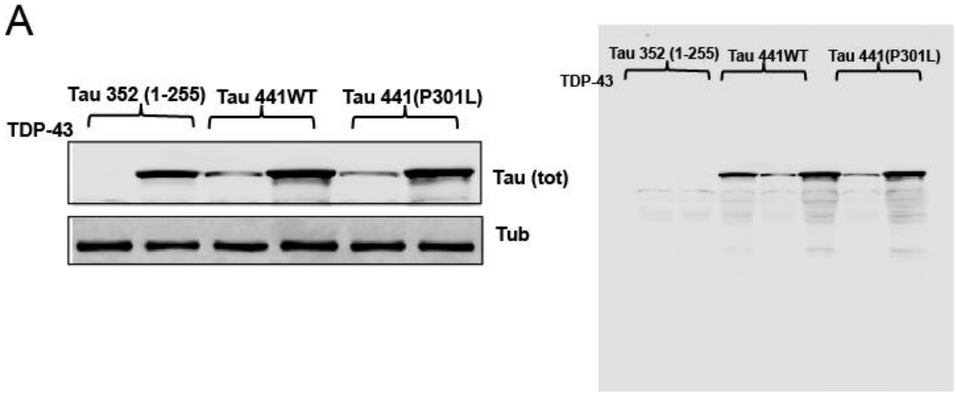

Supplementary File – Uncropped Western Blot Membranes Corresponding to Figures 1–4 and Supplementary Figures. This figure shows the full-length versions of the membranes corresponding to the blots presented in the main and supplementary figures. For reference, the cropped version shown in the final figure is included on the left, and the uncropped membrane is shown on the right. In some cases, lanes were removed because they corresponded to unrelated experiments; however, all relevant sample lanes are shown in full. Please note that for tubulin detection, some membranes were cropped at the time of the experiment (below the 50 kDa marker). In those cases, no further uncropped version exists. All dual detections were performed on the same blot.

The full-length blot corresponding to Figure 1 is currently unavailable, but will be provided if requested.
