# Supplementary material for: 4R-tau isoform induction via TDP-43 in neurons in response to insulin: converging signaling pathways with implications for neurodegenerative disease
Source: Acta Neuropathol Commun. 2025 Dec 24;13:258. doi: 10.1186/s40478-025-02174-x (PMC12729092; doi:10.1186/s40478-025-02174-x)
Supplement: Supplementary file 2 — Supplementary Material 2 [file 40478_2025_2174_MOESM2_ESM.docx]

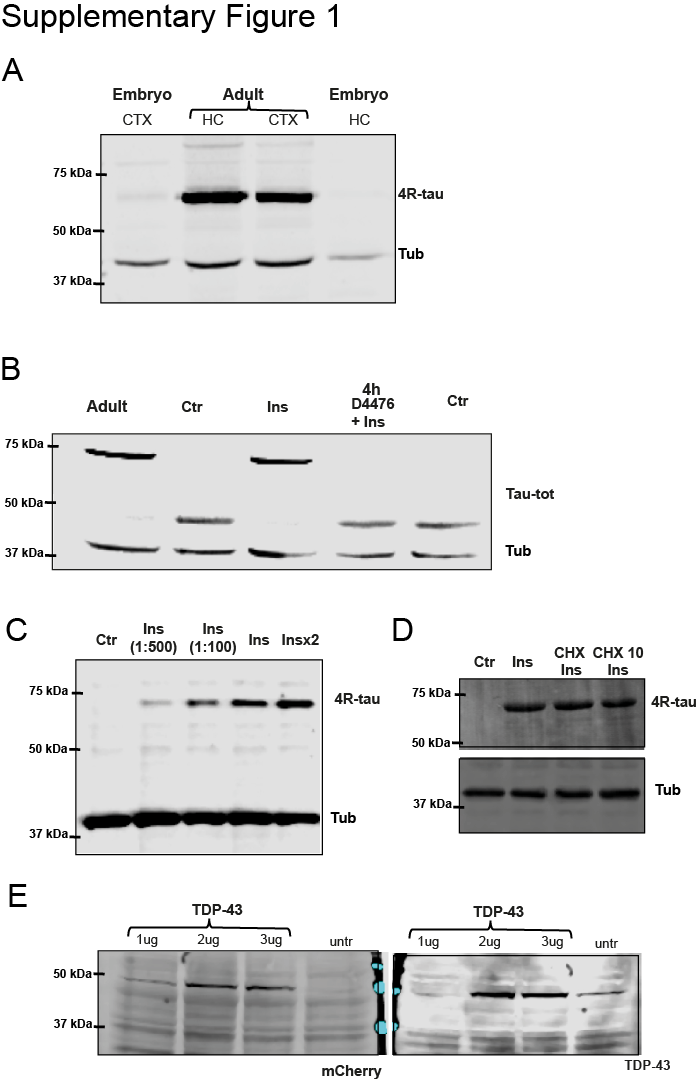


**Supplementary Figure 1**: **Developmental and treatment-dependent detection of Tau isoforms in mice and mCherry-TDP-43 expression in HEK cells** **A)** Western blot showing the absence of 4-repeat tau (4R-tau) during embryonic stages ("Embryo") in primary hippocampal (HC) and cortical (CTX) neuronal cultures prepared at embryonic day 16 (E16) and maintained for 7 days in vitro, compared to its presence in postnatal ("Adult") brain tissue from an 8-month-old mouse**. B)** Western blot detecting total Tau signal in adult brain tissue and in neuronal cultures treated under different conditions. High molecular weight (MW) bands consistent with 4R-Tau are observed in adult and insulin (Ins)-treated cultures, while control (Ctr) and cultures pretreated for 4 h with D4476 prior to Ins exposure show a lower MW band consistent with 3-repeat Tau (3R-Tau). Tubulin (Tub) serves as loading control. **C)**  Cortical cell lysates (Ctr; insulin-treated at Insx2: 100 nM, Ins: 50 nM, Ins 1:100: 0.5 nM, and Ins 1:500: 0.1 nM) showing a dose-dependent increase in 4R-Tau levels (Tubulin as loading control). A clear dose–response effect is observed, with 50 nM insulin producing a robust induction consistent with concentrations commonly used to elicit maximal responses in neuronal cultures. Notably, insulin also increased 4R-Tau expression at near-physiological concentrations (0.1–0.5 nM), comparable to those potentially reached under hyperinsulinemic conditions. **D)** Cortical cultures treated with insulin in the presence or absence of cycloheximide (CHX; 10 or 20 µg/ml, 30 min prior to insulin) showing that insulin’s effect on 4R-Tau expression requires new protein synthesis. **E)** Detection of mCherry-TDP-43 in HEK293 cells. Representative Western blot showing mCherry-TDP-43 in cells transfected with increasing amounts of mCherry-TDP-43 plasmid or left untransfected. The left panel shows detection with an anti-mCherry antibody, and the right panel confirms the fusion protein with an anti–TDP-43 antibody.


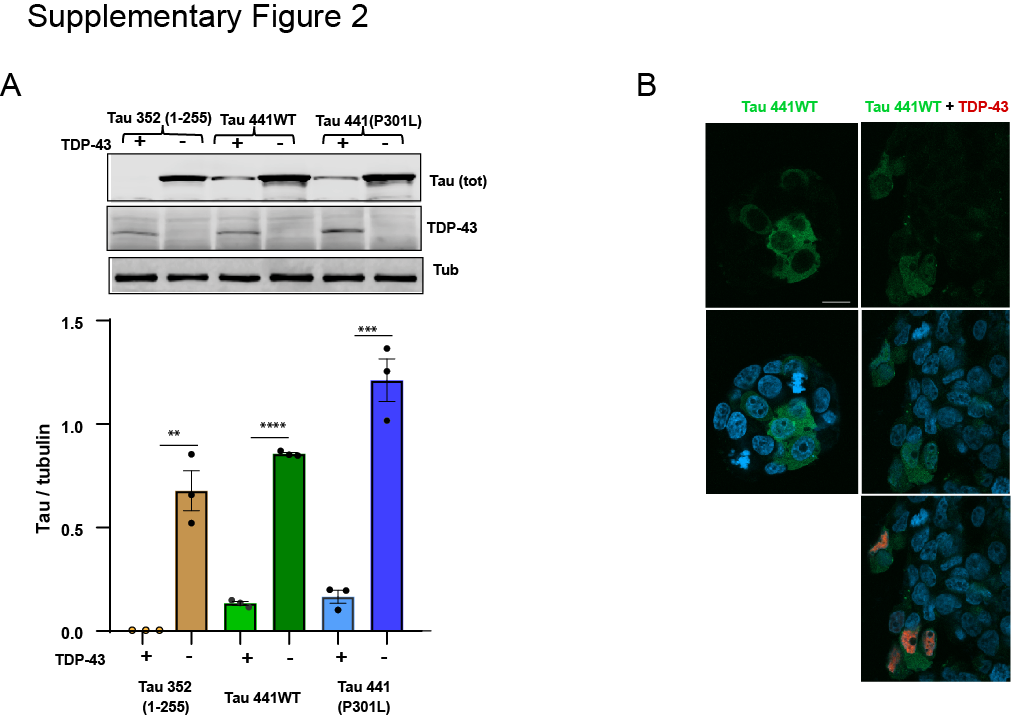


**Supplementary Figure 2. TDP-43 reduces the expression of tau proteins**
**(A)** Representative Western blot (WB) of lysates from HEK cells transfected with plasmids encoding different tau constructs: Tau-352 (1-255) (lacking repeats), Tau-441 wild-type (wt), and Tau-441 carrying the P301L mutation, each fused to fluorescent tags. Co-transfection with TDP-43 plasmids led to a reduction in tau protein levels across all constructs. Quantification below. Data are presented as mean ± SEM; statistical analysis to compare each plasmid (with or without TDP-43) using unpaired Student’s t-test (**p<0.01; *** p<0.001; **** p<0.0001). **(B)** Immunofluorescence images of cells transfected with Tau-441wt alone (green) or co-transfected with TDP-43 (red) given as example. Cells were counterstained with DAPI. Note the reduced green signal in co-transfected cells.


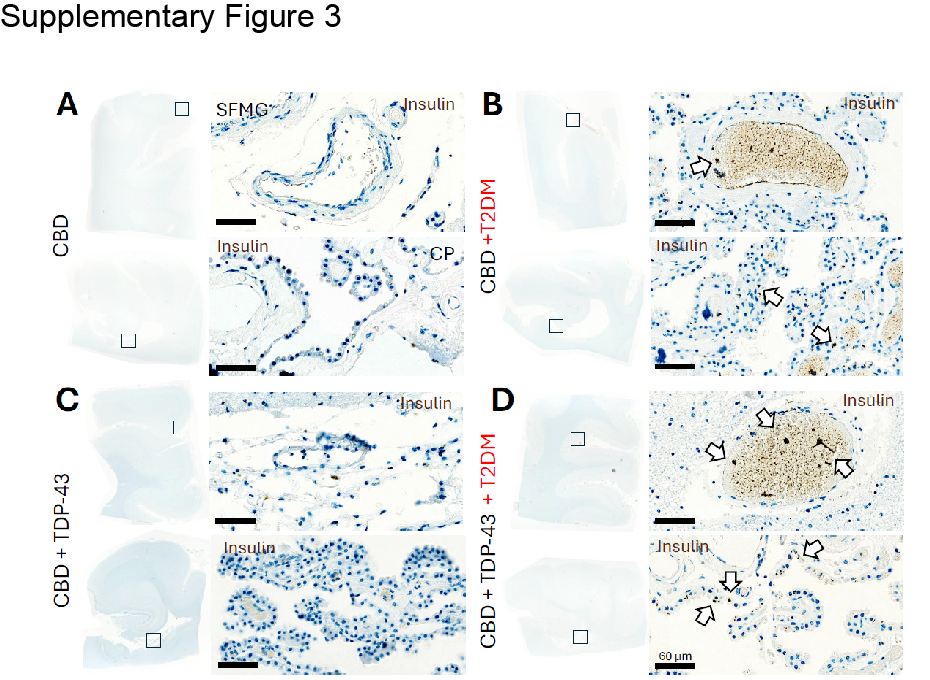


**Supplementary Figure 3 Insulin immunoreactivity in brain samples from patients with corticobasal degeneration (CBD), with or without type 2 diabetes mellitus (T2DM), and with co-occurring TDP-43 pathology.** **A)** Representative images from a CBD case without T2DM showing no detectable insulin staining in cortical vessels of the superior and middle frontal gyrus (SFMG) (top) or in the choroid plexus (CP) (bottom).**B)** In a CBD + T2DM case, insulin immunoreactivity is observed in cortical vessels (top) and in the epithelial lining of the CP (bottom), indicated by black arrows. **C)** A CBD + TDP-43 case shows no insulin labeling in either region. **D)** In a case with combined CBD, TDP-43 pathology, and T2DM, increased insulin immunoreactivity is detected in both cortical vessels and CP (arrows), consistent with elevated systemic insulin levels. Left panels display low-magnification overviews with black squares indicating regions shown at higher magnification on the right. Scale bar = 60 µm
